# Subtype H3N2 Influenza A Viruses: An Unmet Challenge in the Western Pacific

**DOI:** 10.3390/vaccines10010112

**Published:** 2022-01-12

**Authors:** Min Kang, Mark Zanin, Sook-San Wong

**Affiliations:** 1 School of Public Health, Southern Medical University, Guangzhou 510515, China; kangmin@yeah.net; 2 Guangdong Center for Disease Control and Prevention, Guangzhou 511430, China; 3 State Key Laboratory for Respiratory Diseases and National Clinical Research Centre for Respiratory Disease, Guangzhou Medical University, 195 Dongfengxi Road, Guangzhou 511436, China; mark.zanin@gird.cn; 4 School of Public Health, The University of Hong Kong, 7 Sassoon Road, Pokfulam, Hong Kong, China

**Keywords:** influenza A(H3N2), vaccine effectiveness, western pacific, egg-adaptation, novel vaccine platforms

## Abstract

Subtype H3N2 influenza A viruses (A(H3N2)) have been the dominant strain in some countries in the Western Pacific region since the 2009 influenza A(H1N1) pandemic. Vaccination is the most effective way to prevent influenza; however, low vaccine effectiveness has been reported in some influenza seasons, especially for A(H3N2). Antigenic mismatch introduced by egg-adaptation during vaccine production between the vaccine and circulating viral stains is one of the reasons for low vaccine effectiveness. Here we review the extent of this phenomenon, the underlying molecular mechanisms and discuss recent strategies to ameliorate this, including new vaccine platforms that may provide better protection and should be considered to reduce the impact of A(H3N2) in the Western Pacific region.

## 1. Disease Burden of Influenza A(H3N2) Viruses in the Western Pacific

**Figure 1:**
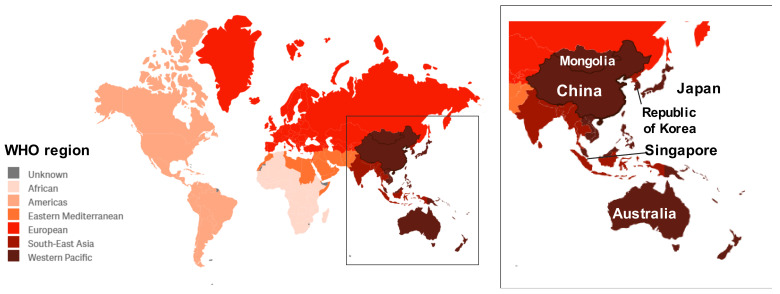
Map of the influenza transmission zones as defined by the WHO. The Western Pacific countries are indicated in dark red. Inset labels the six countries where A(H3N2) were the dominant strain from 2010 week 21 to 2020 week 20. Map downloaded and modified from: https://www.atlasofms.org/map/china/country-classification/who-region# (accessed on 25 November 2021).

Influenza epidemics affect 10–30% of the human population annually. The United States Centers for Disease Control and Prevention (CDC), along with the World Health Organization (WHO), estimated that influenza was associated with 290,000 to 650,000 annual deaths from respiratory diseases from 1999 to 2015 [1], of which approximately 67% occurred in those that are 65 years and older [2]. The highest mortality estimates were in sub-Saharan Africa (2.8–16.5 per 100,000 individuals) and southeast Asia (3.5–9.2 per 100,000 individuals) [1]. The highest proportion of deaths occurred in southeast Asia (68,258–178,049; 25% of total deaths) and countries in the Western Pacific region (67,728–141,436; 25% of total deaths) (Figure 1).

**Figure 2:**
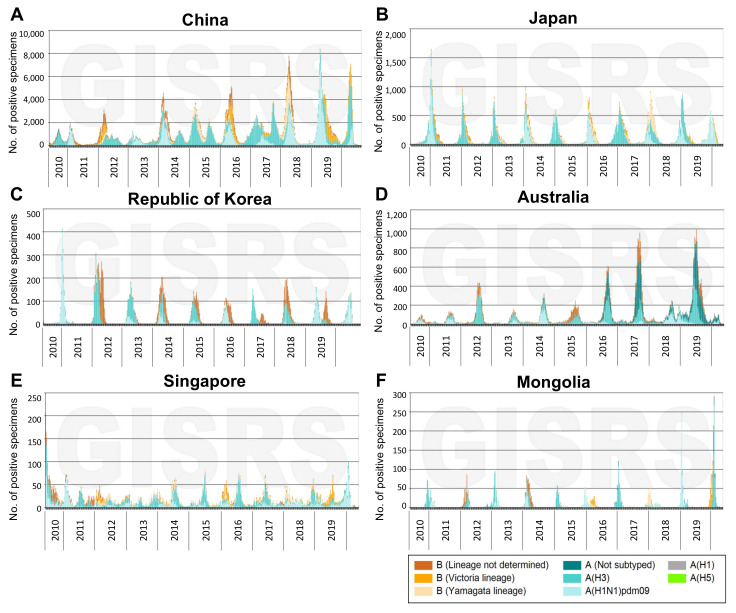
Number of influenza-positive specimens by type and subtype reported in six countries of the Western Pacific region in which A(H3N2) was the dominant strain from 2010 week 21 to 2020 week 20. Specimens were collected in China (**A**), Japan (**B**), the Republic of Korea (**C**), Australia (**D**), Singapore (**E**) and Mongolia (**F**). Data Source: FluNet (www.who.int/flunet, accessed on 11 November 2021).

Subtype H3N2 influenza A viruses (A(H3N2)), which emerged during the 1968 pandemic, are significant contributors to the total influenza disease burden, particularly in the Asian continent. Based on influenza activity by influenza virus types in Asia reported to the Global Influenza Surveillance and Response System (GISRS) between 2011 and 2018, A(H3N2) was responsible for 39.2% of laboratory-confirmed influenza cases in 27 countries across the five Asian influenza transmission zones, compared to 32.5% caused by the two lineages of influenza B viruses (IBVs) and 28.3% caused by A(H1N1) [3]. Specifically, between 2010 and 2020, A(H3N2) accounted for an average of 33% (range 18 to 48%) of cases and was the dominant subtype in 6 of the 13 more populous Western Pacific countries [4] (Table 1 and Figure 2, only countries where A(H3N2) was dominant are shown). This is in contrast to some other populous countries in other WHO regions such as India (South-East Asian), Brazil (Americas), Russia (European) and Pakistan (Eastern Mediterranean), where A(H1N1) viruses were the dominant subtype (data not shown).

Apart from high incidence, A(H3N2) has also been associated with greater disease burden compared to A(H1N1) and IBVs. In Europe, there was a 6- and 3-fold increase in cumulated combined influenza-attributable mortality (excess deaths per 100,000 people) in the elderly during the 2014–2015 and 2016–2017 influenza seasons, respectively, during which A(H3N2) dominated compared to the previous seasons [5]. Greater influenza-like illness and higher influenza-associated morbidity and mortality rates in seasons dominated by A(H3N2) compared with A(H1N1) or IBVs were also reported by countries in the Western Pacific [6,7,8] (Table 2). Furthermore, A(H3N2) has been associated with the highest mortality rates compared to other types/subtypes [5,8,9,10].

## 2. Unsatisfactory Vaccine Effectiveness of A(H3N2) Influenza Viruses

**Figure 3:**
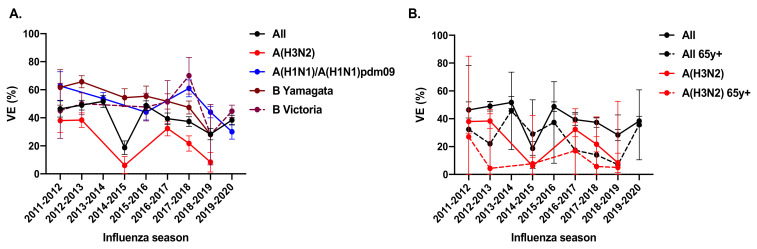
The vaccine effectiveness (VE) against laboratory-confirmed influenza from 2011–2020 in the US by (**A**) strains and (**B**) age against A(H3N2). Data represents the pooled estimates, and error bars represent 95% confidence intervals. Data was obtained from www.cdc.gov/flu/vaccines-work/, (accessed on 12 November 2021).

One of the reasons for the high disease burden associated with A(H3N2) viruses is that the effectiveness of current influenza vaccines against A(H3N2) viruses relative to other types/subtypes is suboptimal [13]. A large systematic review and meta-analysis of test-negative studies evaluating the vaccine efficacy or effectiveness (VE) of egg-based influenza vaccines in the influenza seasons between 2004 to 2015 showed that the pooled global seasonal VE across all ages was 67% (95% CI, 29–85) for A(H1N1), 61% (95% CI, 57–65) for A(H1N1pdm09), 54% (95% CI, 46–61) for type B and only 33% (95% CI, 26–39) for A(H3N2), with higher heterogeneity in VE estimates against A(H3N2) and type B compared to A(H1N1pdm09) [14]. In each age category, pooled VE against A(H1N1pdm09) was 26 to 38 percentage points higher compared to A(H3N2). Among older adults (>60–years old), pooled VE was 63% (95% CI, 33–79) for influenza B, 62% (95% CI, 36–78) for A(H1N1pdm09) and only 24% (95% CI, −6 to 45) for A(H3N2) and was not statistically different from 0. Even in recent years, the VE against A(H3N2) remained lower than other subtypes. Data from the United States between 2011 to 2020 showed that VE against lab-confirmed A(H3N2) influenza cases remained lower in all ages compared to other subtypes, particularly in individuals more than 65 years of age (Figure 3, solid red line and red dotted line) [15,16,17].

Although the elderly are disproportionately affected, low VE can also affect younger age groups. For example, in the US 2018–2019 influenza season, VE against A(H3N2) was only 3% (95% CI, 0–24) for those aged 18–49 years and 0% (95% CI, 0–18) for those aged 50–64 years, compared to 13% (95% CI, 0–48) for those aged ≥65 years [18]. In the same season in Canada, VE against A(H3N2) for those between 20–64 years was calculated to be 32% (95% CI, −119 to 21). Notably, this study also found that the odds of medically attended illnesses of vaccinated people were more than 4-fold higher than those of unvaccinated people among those aged between 35–54 years, which might be due to the disruption of pre-existing immunity to influenza by egg-based vaccines [19].

These data from the US suggested that VE against A(H3N2) has been generally poorer since 2011. To understand if this phenomenon extends to countries in the Western Pacific region, we conducted a non-exhaustive literature search and summarized the studies reporting influenza VE in this region (Table 3). There were 22 studies from 7 countries reporting VE between the period of 1997 to 2020. Except for a cohort study in children in Suzhou, China and a meta-analysis from Japan, all were case-controlled, test-negative observational studies. While differences in VE against A(H3N2) versus other strains rarely met statistical significance, 16 studies across different influenza seasons reported lower VE against A(H3N2) compared to other subtypes, including 7 that were conducted during A(H3N2) dominant seasons.

In China, VE is usually reported by individual studies for one or two influenza seasons in localities with relatively higher influenza vaccine coverage (i.e., Beijing, Hong Kong and Suzhou). Among the 13 studies that reported VE against A(H3N2), 9 reported that VE against A(H3N2) was lower than the overall VE and 3 studies reported negative VE against A(H3N2) [18,20,21,22,23,24,25,26,27,28,29,30,31] (Table 3). In Japan, a meta-analysis of 143 studies from 1997–2018 showed that “VE reported during seasons of H1 virus dominance was slightly higher (22.0%, 95% CI: −25.9–51.7) than seasons of H3 (19.3%, −13.3–42.6) or mixed/B virus subtype circulation (14.6%, −13.7–35.9)” [32]. In Korea, although VE was frequently reported, A(H1N1) and A(H3N2) were not distinguished. One interim report during the 2016–2017 season estimated the VE against A(H3N2) to be 52% (95% CI: −147%, 6%) [33]. In Australia, with the exception of 2013, the VE against A(H3N2) from 2010 to 2017 was consistently lower compared to the VEs against A(H1N1) or B-lineages [34,35,36,37]. In Singapore, one study in military personnel reported lower VE against A(H3N2) versus other strains [38], although another study in the elderly in long term care facilities reported lower VE against A(H1N1) pandemic 2009 strains compared to A(H3N2) [39]. In New Zealand, a four-year study by the Southern Hemisphere Influenza and Vaccine Effectiveness Research and Surveillance team reported lower VE against A(H3N2) for severe influenza outcomes than against A(H1N1) or B-lineage [40]. VE results in Cambodia, Laos, Malaysia, Mongolia, Papua New Guinea, Philippines and Vietnam were unavailable, presumably as improving vaccine coverage rates remains the main focus of healthcare authorities and researchers in these countries.

## 3. Reasons for Low VE against A(H3N2) Influenza

### 3.1. Antigenic Drift of A(H3N2) Viruses

Besides population immunity, another critical determinant of influenza VE is the antigenic match between the vaccine strain and the circulating strain. Vaccine mismatch can occur due to either antigenic drift or egg adaptation during vaccine preparation. The strain composition of the influenza vaccine for the upcoming influenza season is based on the WHO recommendation derived from influenza surveillance data collected through the GISRS network. This recommendation is made 6–12 months prior to the influenza season, mainly to allow for egg-based vaccine production [41].

Antigenic drift happens when mutations occur on the antigenic sites of the hemagglutinin (HA) and neuraminidase (NA) proteins of circulating influenza viruses that lead to poor antibody recognition. [42]. The globular head of HA is immunodominant with five major antibody binding sites in the A(H3N2) subtype. Amino acid substitutions that occur at these key positions can result in the loss of antibody recognition. One of the known mechanisms of immune evasion is through the acquisition of N-linked oligosaccharides (glycans) that shield the antigenic sites. A(H3N2) viruses circulating in recent years possess six to seven glycosylation sites in the HA head compared to only two glycosylation sites in the HA head of A/Hong Kong/1/1968 (H3N2), the earliest known human A(H3N2) pandemic virus. The more rapid antigenic evolution of A(H3N2) viruses compared to other subtypes of influenza virus appears to account for the dominance of seasonal A(H3N2) influenza after the 1968 pandemic. The antigenic evolution rate of A(H3N2) was approximately 17 times higher than that of A(H1N1pdm09) and approximately 5–6 times higher than that of IBVs [43].

### 3.2. Egg-Adaptation of A(H3N2) Viruses

Egg-based influenza vaccine manufacturing accounts for a vast majority of the total influenza vaccines supplied globally each year. Egg-adaptation of A(H3N2) viruses has been recognized in recent years as a major reason for low VE and vaccine mismatch [13]. Mammalian-adapted viruses such as A(H3N2) may encounter strong selection pressure when reproducing in avian cells because of biological differences between human and avian cells. For instance, influenza virus HA binds to sialic acid residues on the surface of host cells, which are predominantly α-2,6 type in the human upper respiratory tract and α-2,3 type in eggs. The difference dampens human influenza viral binding and growth in eggs [44] and selects variants with enhanced binding to avian-type receptors [44,45,46,47]. Consequently, some of these substitutions may change the antigenicity of egg-adapted influenza viruses and decrease the neutralizing antibody responses against prototype viruses [13].

The first study to link low A(H3N2) VE to egg adaptation and not antigenic drift was Skowronski et al. [48]. During the 2012-2013 influenza season, the genotypic and phenotypic match between the WHO-recommended A/Victoria/361/2011 (H3N2) prototype and A(H3N2) field isolates was proved by sequencing HA antigenic sites and by hemagglutination-inhibition (HI) assays. However, most field isolates of A(H3N2) were antigenically distinct from egg-adapted strains employed in vaccine production. The egg-adapted vaccine strain had three amino acid substitutions at antigenic sites B (H156Q, G186V) and D (S219Y) compared to the WHO-recommended prototype. Compared with the egg-passaged strain, between 8- to 32-fold reductions in HI antibody titer occurred in all, but one A(H3N2) field isolate. Therefore, mutations in the egg-adapted vaccine strain, in addition to antigenic drift in circulating viruses, are contributing to the poor VE against A(H3N2) strains.

#### 3.2.1. Molecular Basis of A(H3N2) Egg Adaptation

Egg adaptation can result in mutations in the receptor binding sites (RBS) or alterations to the glycosylation profile of the HA head, leading to impaired antigenicity and binding avidity. Adaptive mutations can emerge after as few as three passages of human A(H3N2) viruses [49,50]. Recent investigations of the HA L194P and K160T substitutions have provided a potential mechanism of how egg-adaptive mutations can be linked to low VE of A(H3N2) vaccine strains. For example, the substitution HA L194P was prevalent in the WHO-recommended A(H3N2) vaccine strains after passaging in eggs during the 2008–2010, 2016–2018 and 2018 influenza seasons. This replacement of Leucine by Proline alters the mobility of the 190-helix and neighboring regions in antigenic site B, resulting in reduced binding affinity of RBS-targeted neutralizing antibodies by three orders of magnitude [47].

Loss of a glycosylation site has been identified as another egg-adaption in A(H3N2), leading to an antigenic mismatch. A K160T HA mutation that emerged during the 2014–2015 influenza season, highly prevalent in the 2016–2017 influenza season, was predicted to introduce an N-linked glycosylation site in the antigenic site B of HA. However, due to egg adaptation, 2016–2017 egg-based A(H3N2) vaccine strain possessed a T160K HA reversion mutation, which abolished the glycosylation site on antigenic site B. Antigenic testing showed that antibodies elicited in ferrets and humans exposed to the egg-adapted 2016–2017 A(H3N2) vaccine strain poorly neutralized the circulating clade 3C.2a A(H3N2) viruses. Meanwhile, antibodies elicited in ferrets infected with a circulating A(H3N2) virus, and humans vaccinated with baculovirus-expressed H3 antigen lacking the egg-adaptation mutation but still possessing the glycosylation site were able to neutralize the glycosylated clade 3C.2a A(H3N2) virus [51]. Therefore, differences in glycosylation between A(H3N2) egg-adapted vaccine strains and circulating strains likely contributed to the reduced VE during the 2016–2017 influenza season.

#### 3.2.2. Consequence of Egg Adaptation on Immunogenicity and VE

Katz et al., first reported differences in immunogenicity with egg-grown versus cell-grown variants [45]. After propagation in embryonated chicken eggs compared to Madin-Darby canine kidney (MDCK) cells, a single amino acid substitution in the HA of A/Memphis/12/1985 (H3N2) resulted in an antigenically distinct variant distinguishable by immune ferret serum. The egg-propagated virus also elicited lower serum HA-inhibiting and neutralizing antibody titers compared to the MDCK-propagated virus and resulted in reduced protective efficacy in ferrets [50]. In another study, a high-growth reassortant A/Victoria/210/2009 (H3N2) (X-187) propagated in eggs was used as the vaccine strain against A(H3N2) viruses from the 2010-2011 influenza season in Japan. Post-infection ferret antisera could not neutralize epidemic strains of MDCK-propagated A(H3N2) viruses, and post-vaccination human serum showed low cross-reactivity to both egg- and MDCK-passaged A(H3N2) viruses [52]. Finally, the WHO, through its network collaborating centers, reported that ferret antisera raised against the egg-propagated A/Victoria/361/2011 (H3N2), the strain recommended for 2012-2013 influenza season, recognized MDCK-passaged virus isolates poorly, with an 8-fold or greater reduction in titers compared with the egg-grown virus [53]. Bioinformatics studies revealed that repeated convergent evolution at certain HA codons drives egg adaptation of A(H3N2). Chen et al., calculated the relationship between egg-passage adaptations and the efficacy of A(H3N2) influenza vaccines. Using large scale sequence data, they found that the more mutations that accumulate at codons associated with egg-passage, the lower the VE against A(H3N2) viruses [54]. Collectively, the studies show that egg-adaptation of A(H3N2) has impacted VE over multiple influenza seasons and geographic locations.

#### 3.2.3. Influence of Pre-Existing Immunity to Influenza Vaccine Responses

Prior vaccination can interfere with subsequent vaccination, depending on interactions between the vaccine strains of the previous and current year and the circulating strain [55]. For example, vaccine components were conserved in the 2013–2014 and 2014–2015 seasons in Canada, while the dominant circulating A(H3N2) strain was mismatched in 2014–2015. As a result, VE against A(H3N2) in the 2014-2015 influenza season was lower among individuals vaccinated in both the 2013–2014 and 2014–2015 seasons (VE = 32%, [95% CI, –75% to 0%]) compared to those vaccinated in the 2014–2015 season alone (VE = 53%, [95% CI, 10%–75%]) [56]. Another example is the 2018–2019 season when VE against A(H3N2) was lower in individuals aged 35–54 years compared to individuals more than 65-years-old and unvaccinated people. The 35–54-year-old age group, in their childhood, were exposed to the 1968 pandemic A(H3N2) strain harboring a serine at HA position 159, the same as circulating clade 3c.3a viruses but mismatched to vaccine 3c.2a strains [19].

Pre-existing immunity can also be influenced by egg adaptation. Liu et al., investigated how egg adaptation-mediated vaccine mismatch impacted antibody responses across different age groups over three consecutive influenza seasons (2016–2019). Egg-adapted vaccine strains bearing T160K, D225G, and L194P mostly elicited antibodies against egg-adapted epitopes instead of those in wild type A(H3N2) viruses in immunologically naive children, but broader responses were observed in children previously primed by infection. Repeated boosting with an egg-adapted vaccine led to significantly reduced antibody responses in the elderly to wild type viruses [57]. These studies suggest that pre-existing immunity brought about by infection or vaccination may explain the link between age and VE variation and that egg-adaptive vaccine mismatches may negatively affect imprinted immunity [19]. However, other studies found no impact on protection from prior infection or repeated vaccination [58,59,60,61]. Mclean et al., found no association between prior vaccination and reduced VE in children receiving inactivated or live attenuated influenza vaccines [55]. Another study by Nichols et al., also demonstrated no significant overall associations between prior season vaccinations and lower VE, although they observed non-significant trends of decreased VE in A(H3N2)-dominant seasons among those repeatedly vaccinated [62]. The heterogeneity observed in these studies suggests that the impact of pre-existing immunity is not fully understood and is likely influenced by the seasonal variations of the circulating viruses.

## 4. Vaccines beyond Egg-Based Formula for Better Protection against Influenza

Although egg-based influenza vaccines remain the most widely used, several novel vaccine platforms show potential to overcome limitations associated with egg-based vaccines. This new generation of egg-free vaccines are developed with speed and ease of manufacturing in mind. We discuss several egg-free influenza vaccines that have been approved by the US Food and Drugs Administration (FDA), European Medicines Agency (EMA) and several health authorities in the Western Pacific region, namely Australia and Hong Kong.

### 4.1. Cell-Based Vaccine

Cell-based influenza vaccines produced using viruses grown in cultured cells of mammalian origin avoid egg-adaptive mutations. Other advantages include ease of supply, as cell lines can be easily stored and exponentially expanded, and avoiding issues of allergies to egg proteins. The greater standard of sterile control over cell cultures and raw materials also significantly lowers the risk of microbial contamination [63]. Cell-based influenza vaccine appeared as early as 2001, and the current widely distributed cell-based influenza vaccine Flucelvax (Sequirus) was approved by the FDA in 2012 [63]. In its registration trial, during the A(H1N1) and IBV co-dominant 2007–2008 season, the cell-based vaccine achieved 69.5% VE against all influenza strains for all culture-confirmed cases; VE against A(H3N2), at 75.6%, was approximately 1.5-fold higher compared to that of the egg-based vaccine, at 49.3% [64]. In the A(H3N2)-dominant 2017-2018 influenza season in the US, the cell-based vaccine reduced the influenza-related hospitalization rate by 10% (95% CI, 7–13) compared to the egg-based vaccine [65].

However, viral adaptation can also occur during cell passages, although mutations that affect antigenicity may occur less often compared to egg culture [66,67]. Furthermore, the immunogenicity of cell-based vaccines is similar to egg-based vaccines, as both stimulated similar antibody responses in the 2017–2018 and 2018–2019 influenza seasons [68,69] and provided similar levels of protection during 2018–2019 and 2019–2020 influenza seasons among those aged 65 years and above in the US [70,71].

### 4.2. Recombinant HA (rHA) Vaccine

Recombinant influenza vaccines rely on molecular methods to synthesize vaccine antigens. It precludes egg- or cell-adaptive mutations as the target HA is synthesized based on the specific HA-sequence and expressed using non-egg-based systems. Manufacturing requires 6 to 8 weeks compared to the 6 months needed for egg-grown vaccines [72]. The first rHA vaccine, Flublok, was licensed by the FDA in 2013 for adults aged 18 and older. The numerous studies conducted using Flublok have demonstrated its immunogenicity and efficacy across all ages. Flublok is formulated at 45 μg per HA, which is three times the amount of HA contained in the standard-dose of inactivated vaccines [73,74] and has a demonstrated VE of 44.6% (95% CI, 18.8–62.6%) in preventing culture-confirmed influenza during the 2007–2008 influenza season [75]. Importantly, Flublok also appears to offer potential advantages during seasons of antigenic mismatch [76]. Dunkle et al., measured the efficacy of Flublok in individuals 50 years of age or older in the US during the 2014–2015 influenza season when A(H3N2) viruses predominated, and the vaccine influenza strains were antigenically mismatched from the circulating virus [77]. The laboratory-confirmed influenza attack rate was 2.2% among Flublok recipients, and 3.1% among standard dose egg-based inactivated vaccine recipients, which is a 30% (95% CI, 10 to 47) reduction in influenza attack rate in the Flublok recipients.

Flublok has also been shown to improve immunogenicity in comparison to egg- or cell-grown vaccines. Gouma et al., analyzed the immunogenicity of sera collected from 85 individuals 18–49 years of age vaccinated with either egg-based vaccine, cell-based vaccine or recombinant protein-based vaccine during the 2017–2018 influenza season [68]. Geometric mean titers (GMTs) of neutralizing antibodies against wild-type Clade 3c.2a and Clade 3c2.a2 A(H3N2) viruses in those vaccinated with Flublok were 3.9 to 4.3-fold higher than those who received the egg-based or cell-based vaccines. Flublok elicited similar neutralizing antibody titers against the wild-type A(H3N2) viruses as the high dose egg-based vaccine (which contained 4-times the standard dose), and both were significantly better than those who received the standard dose of egg-based vaccine. A larger fraction of Flublok vaccinees seroconverted to wild-type A(H3N2) viruses (52% to 3c.2a virus and 61% to 3c2.a2 virus) compared to those that received the high-dose of egg-based vaccine (38% to 3c.2a virus and 38% to 3c2.a2 virus). Another randomized clinical trial found that the Flublok vaccine elicited statistically significantly higher seroconversion rates (61%) against A(H3N2) viruses compared to standard dose egg-grown vaccine (44%) in individuals 50–64 years of age [78].

The enhanced immunogenicity of Flublok compared to standard egg-based vaccines were also observed in older adults. In individuals 65–74 years of age [79], Flublok elicited a greater mean fold-rise (2.0, 95% CI 1.7–2.5) against the vaccine strain relative to a high-dose egg-based vaccine (1.6, 95% CI 1.3–1.8) and a greater mean fold-rise (2.9–3.5) against a diverse clade of circulating viruses, including the 2019–2020 vaccine strain, compared to the high-dose egg-based vaccine (1.3–1.6) and adjuvanted egg-based vaccine (1.6–1.7).

A randomized, controlled clinical trial was also conducted to evaluate the immunogenicity of an MF59-adjuvanted trivalent vaccine, high-dose trivalent vaccine and Flublok in the elderly (aged 65–82 years) during the 2017–2018 influenza season in Hong Kong [80]. The post-vaccination mean titers in participants that received Flublok was 2.57-fold higher compared to the standard dose of cell-propagated A(H3N2) vaccine and significantly greater compared to the MF59-adjuvanted egg-based vaccine (1.43-fold) and the high-dose egg-based vaccine (1.33-fold).

Another rHA vaccine, NanoFlu, consists of baculovirus-expressed and purified HA homotrimers assembled into nanoparticles and delivered with a novel saponin-based adjuvant. NanoFlu has shown promising results and granted an accelerated approval pathway by the FDA [81,82]. In its Phase III trial, it showed immunological non-inferiority to IIV4 for all four strains tested and, in particular, a significantly higher GMT ratio of 1·19 (95% CI: 1·11 to 1·27), and higher seroconversion rates (7·3%, (95% CI: 3.6 to 11.1) to A/Kansas 14/2017 (H3N2) [82].

### 4.3. Other Technologies for Non-Egg-Based Influenza Vaccines

New technology and manufacturing platforms, including structure-based antigen design, virus-like nanoparticles, gene- and vector-based technologies and potentially universal influenza vaccines, show potential to overcome the limitations of the current egg-based vaccines [41,74]. Nucleic acid platforms, such as DNA and mRNA, are attractive approaches due to their ease of production, high quality, short lead-time and adaptability to encode various antigens [74]. mRNA vaccines demonstrated approximately 95% efficacy against SARS-CoV-2 virus infection in a Phase III trial and have shown remarkable effectiveness in reducing transmission [83]. The huge success of mRNA vaccines against SARS-CoV-2 infection encourages further application in preventing influenza, offering rapid production, reduced lead time and better antigenic matching to circulating strains. mRNA vaccines were first developed against influenza in 2012, serving as precedent to the current success against COVID-19 [84]. mRNA vaccines against influenza have since demonstrated early promise in animal studies and Phase I clinical trials [85,86,87,88,89].

There is a push to improve current influenza vaccine platforms to a target of at least 90% effectiveness for a duration of 3–5 years [74]. A ‘universal’ influenza vaccine, primarily based on constructs that target conserved epitopes amongst the different influenza subtypes, such as those located in the stem/stalk domain of HA, is expected to elicit broad reactivity against both seasonal and emerging strains of influenza viruses [90,91]. Once validated and tested, these new platforms and methods may prove to be the future of influenza vaccinations. However, for the time being, maximizing the potential of currently available improved vaccines remains the most viable and practical approach to ease the burden of A(H3N2) influenza.

## 5. Summary

While egg-adaptation is not restricted to A(H3N2) [92,93] and reflects a general problem of egg-based influenza vaccines, existing literature indicates that this is a greater problem for A(H3N2) influenza strains [94,95]. Poor VE against A(H3N2) viruses that are antigenically mismatched to vaccine candidate strains are clearly documented in the US population and appears to also affect countries with some vaccine coverage in the Western Pacific region. Influenza vaccination in this region presents challenges due to diverse transmission patterns, immunization practices and public health policies, which have not been covered here. Although overall influenza vaccine coverage is still relatively poor in this region, vaccine awareness in the COVID-19 era may change attitudes and improve influenza vaccine uptake in coming years. Therefore, vaccination platforms that can be accessible to large populations and offer protection beyond a single influenza season are important considerations. Whilst influenza activity post-COVID-19 has been low, recent reports indicate A(H3N2) viruses have returned to pre-COVID levels of activity in some Southeast Asian countries [96]. Given the higher disease burden and the purported importance of some countries in the Western Pacific region in the evolution and global spread of A(H3N2) viruses [97,98,99,100,101], there is a need for a better assessment of VE. In addition, the value of next-generation influenza vaccines should also be considered in reducing the impact of A(H3N2) in this region.

## Figures and Tables

**Table 1:** Frequency of laboratory-confirmed influenza cases by influenza virus type and subtype captured by FluNet from 2010 week 21 to 2020 week 20 in 13 countries of the Western Pacific region with populations greater than 1 million ^a^.

		Percentage of Cases by Influenza Virus Type/Subtype			
Country ^a^	10-Year Total Number of Influenza Positive Reported	A(H3)	A(H1, H1N1pdm09)	A(Unsubtyped)	B Total
**China**	679,983	**40.8%**	27.0%	0.9%	31.3%
**Japan**	82,710	**45.4%**	29.9%	0.1%	24.6%
**Australia**	53,577	**34.1%**	15.2%	29.6%	21.2%
**Republic of Korea**	18,444	**39.6%**	25.9%	0.0%	34.6%
New Zealand	17,235	25.9%	15.1%	25.9%	33.1%
**Singapore**	10,985	**38.4%**	29.8%	0.8%	31.0%
Vietnam	6955	32.7%	32.8%	0.0%	34.5%
Philippines	6105	24.9%	26.1%	6.2%	42.8%
**Mongolia**	5677	**48.1%**	25.1%	2.5%	24.3%
Cambodia	4603	31.4%	31.2%	0.0%	37.4%
Laos	4512	33.5%	27.8%	0.1%	38.6%
Malaysia	3322	17.8%	31.1%	14.2%	36.9%
Papua New Guinea	261	19.5%	46.4%	5.0%	29.1%
Total	894,369				
Mean, % (95% CI)		33.2% (27.6–38.9)	27.9% (23.2–32.7)	6.6% (0.4–12.7)	32.2% (28.5–36.0)

^a^ Data Sources: FluNet (www.who.int/flunet, accessed on 11 November 2021) and Global Influenza Surveillance and Response System (GISRS) (https://apps.who.int/flumart/Default?ReportNo=10, accessed on 12 November 2021). Bold indicates countries and their corresponding percentages in which A(H3N2) was the dominant strain.

**Table 2:** Severity of outcomes associated with influenza cases by virus type, region and year.

Category	Years	A(H3N2) *,**	A(H1N1) *	B-Lineage *	Region	Ref.
Incidence -Influenza-like illness consultations per 1000 person-year	2010–2015	**0.7** (0.4–0.9)	0.5 (0.3–0.7)	0.3 (0.0–0.5)	China	[6]
Hospitalization -Rates per 100,000 person-years	2010–2011	**55**	33	26	China	[7]
-Risk ratio to A(H1N1)	2009–2011	**1.8** (1.3–2.6)	1		Hong Kong	[8]
Death -Excess mortality rates per 100,000 person-seasons	2010–2015	**2.6** (2.4–2.8)	1.6 (1.5–1.7)	2.3 (2.1–2.5)	China	[11]
-Excess mortality rates per 100,000 person-seasons	1998–2009	**6.88** (4.26–9.37)	1.6 (−0.34–3.34)	2.5 (−0.51–5.33)	Hong Kong	[9]
-All-cause death risk ratio	2009–2011	**2.6** (1.8–3.7)	1		Hong Kong	[8]
-Respiratory death risk ratio	2009–2011	**1.5** (1.0–2.1)	1		Hong Kong	[8]
-All-cause death risk ratio	1996–2003	**1.04** (1.02–1.05)	1	1.01 (1.00–1.02)	Singapore	[12]
-Respiratory death risk ratio	1996–2003	**1.08** (1.04–1.12)	1	1.00 (0.97–1.03)	Singapore	[12]
-Excess mortality rates per 100,000 person-seasons	2009–2016	**8.66** (5.88–11.35)	5.99 (3.41–8.46)	4.77 (1.04–8.24)	Hong Kong	[10]

* 95% confidence interval in the parentheses. ** Numbers in bold represent statistically different from A(H1N1) or other references.

**Table 3:** Summary of studies reporting vaccine effectiveness (VE) against A(H3N2) during 2010–2020 in several Western Pacific countries. Studies conducted during which A(H3N2) was the dominant circulating subtype are highlighted in bold, and the relatively low A(H3N2) VE amongst subtypes are underlined. All studies, except the meta-analysis from Japan, used test-negative, case-control study designs.

Season	Region/City	Overall VE % (95% CI ^a^)	VE % (95% CI) against			Circulating Subtype ^b^	No. Positives	Study Population	Outcome ^c^	Ref.
			A(H3N2)	A(H1N1)	B					
2012–13	Beijing, China	52 (12, 74)	43 (−30, 75)	59 (8, 82)	Not provided	A(H1N1)	695	All ages	Medically attended ILI	[20]
2013–14	Beijing, China	32 (−48, 69)	22 (−253, 83)	59 (−79, 90)	−20 (−239, 58)	A(H1N1)	133	60 years and older	Medically attended ILI	[21]
2013–14	Beijing, China	47 (−20, 77)	60 (−110, 92)	Not provided	42 (−60, 79)	A(H1N1)	353	All ages	Influenza-associated hospitalization	[22]
2014–15		5 (−53, 41)	28 (−42, 63)	Not provided	−32 (−154, 32)	A(H3N2)				
**2014–15**	**Beijing, China**	**−18 (−49, 6)**	**−*25* (*−70, 8*)**	**Not provided**	**−8 (−50, 23)**	**A(H3N2)**	**3434**	**All ages**	**Medically attended ILI**	[23]
2015–16	Beijing, China	8 (−16, 27)	54 (16, 74)	18 (−38, 52)	−7 (−38, 18)	A(H1N1) and B	2969	All ages	Medically attended ILI	[18]
2015–16	Beijing, China	−38 (−103, 6)	−5 (−108, 47)	−62 (−212, 16)	−45 (−153, 16)	A(H1N1) and B	356	All ages	Influenza-associated hospitalization	[24]
**2016–17**	**Suzhou, China**	**21 (−42, 56)**	***1 (* *−86, 47)***	**Not provided**	**63 (−65, 92)**	**A(H3N2)**	**70**	**Children 36–72 months old**	**Influenza infection**	[25]
**2016–17**	**Beijing, China**	**25 (0, 43)**	***2* (−*35, 29*)**	**54 (22–73)**	**Not provided**	**A(H3N2)**	**2626**	**All ages**	**Influenza-associated outpatient visits**	[26]
**2016–17**	**Beijing, China**	**69 (51, 81)**	**73 (53, 85)**	**60 (−15, 86)**	**Not provided**	**A(H3N2)**	**176**	**School Children 6–19 years old**	**Influenza infection**	[27]
**2016–17**	**Beijing, China**	**33 (−22, 63)**	**30 (−30, 62)**	**Not provided**	**Not provided**	**A(H3N2)**	**145**	**60 year and older**	**Influenza-associated hospitalization**	[28]
2017–18		5 (−72, 47)	−38 (–294, 52)	29 (−93, 74)	4 (−114, 56)	A(H1N1) and B	149			
2009–13	Hong Kong, China	62 (43, 74)	37 (−26, 68)	72 (39, 87)	69 (42, 83)	N/A	451	Children 6m–17 years old	Influenza-associated hospitalization	[29]
2017–18	Hong Kong, China	59 (41, 72)	41 (−60, 82)	86 (66, 95)	54 (35, 75)	N/A	467	All ages	Primary care visits	[30]
2019–20	Hong Kong, China	65 (46, 78)	12 (−80, 57)	74 (54, 85)	85 (30, 97)	N/A	198	Children 6m–17 years old	Influenza-associated hospitalization	[31]
1997–2018	Japan	19 (2, 33)	19 (−13, 43)	22 (−26,52)	15 (−14, 36)	N/A	(Meta-analysis from 143 studies)			[32]
**2016–17**	**Korea**	**−36 (−115, 14)**	**−*52* (*−147, 6)***	**Not provided**	**Not provided**	**A(H3N2)**	**216**	**Adults**	**Influenza infection**	[33]
2010	Western Australia	68 (35, 85)	3 (−495, 84)	80 (41, 93)	66 (1,89)	A(H1N1)	448	All ages	ILI GP visits	[34]
2011		52 (1, 77)	−55 (−386, 5)	71 (15, 90)	85 −30, 98)	A(H1N1)	351			
**2012**		**49 (30, 63)**	**46 (21, 63)**	**8 (−868, 91)**	**54 (26, 71)**	**A(H3N2)**	**1161**			
**2012**	**Australia**	**38 (24, 49)**	***30 (14, 44)***	**54 (−28, 83)**	**56 (37, 70)**	**A(H3N2)**	**1462**	**All ages**	**ILI GP visits**	[35]
**2013**		**60 (45, 70)**	**67 (39, 82)**	**59 (33, 74)**	**57 (30, 73)**	**A(H1N1) and A(H3N2)**	**441**			
2014		44 (31,55)	26 (1, 45)	55 (39, 67)	54 (21, 73)	A(H1N1)	891			
**2015**	**Australia**	**54 (42, 63)**	***44 (21, 60)***	**79 (33, 93)**	**58 (45, 68)**	**A(H3N2) and B**	**857**	**All ages**	**Medically attended ILI**	[36]
**2017**	**Australia**	**33 (17, 46)**	***10* (−*16, 31*)**	**50 (8, 74)**	**57 (41, 69)**	**A(H3N2)**	**1060**	**All ages**	**Medically attended ILI**	[37]
2010–13	Singapore	not provided	33 (−4, 57)	84 (78, 88)	84 (79, 86)	N/A	1198	Military Adults	Influenza infection	[38]
**2017**	**Singapore**	**40 (−12, 68)**	**57 (6, 80)**	**−43 (−312, 50)**	**Not provided**	**A(H3N2)**	**118**	**Seniors in Long-term care facilities**	**Influenza infection**	[39]
2012–15	Auckland, NZ	37 (23, 48)	26 (5, 42)	42 (14, 61)	49 (30, 63)	N/A	842	Adults	ICU admission and Severe Disease	[40]

^a^ CI: Confidence Interval. ^b^ Dominant subtype in circulation data (country-level) obtained from GISRS (https://apps.who.int/flumart/Default?ReportNo=10, accessed on 12 November 2021). ^c^ ILI: Influenza-like Illness.

## Data Availability

Data used to produce the figures and tables in this review is available upon request.
